# Fatal Fournier’s gangrene caused by *Clostridium ramosum* in a patient with central diabetes insipidus and insulin-dependent diabetes mellitus: a case report

**DOI:** 10.1186/s12879-018-3280-9

**Published:** 2018-08-02

**Authors:** Noriyoshi Takano, Midori Sasaki Yatabe, Junichi Yatabe, Masaaki Kato, Daisuke Sueoka, Shigekazu Iguchi, Atsushi Yoshida, Yutaka Uzawa, Ken Kikuchi, Kimitaka Tani, Shinpei Ogawa, Michio Itabashi, Masakazu Yamamoto, Daisuke Watanabe, Takashi Ando, Satoshi Morimoto, Atsuhiro Ichihara

**Affiliations:** 1 Department of Medicine II, Endocrinology and Hypertension, Tokyo Women’s Medical University, 8-1 Kawada-cho, Shinjyuku-ku, Tokyo, 162-8666 Japan; 2 Medical Training Center for Graduates, Tokyo Women’s Medical University, Tokyo, Japan; 3 Department of Infectious Diseases, Tokyo Women’s Medical University, Tokyo, Japan; 4 Department of Gastroenterology, Tokyo Women’s Medical University, Tokyo, Japan

**Keywords:** Fournier’s gangrene, Necrotizing fasciitis, Opportunistic infection, *Clostridium ramosum*

## Abstract

**Background:**

*Clostridium ramosum* is a generally non-pathogenic enteric anaerobe, and Fournier’s gangrene is a rare necrotizing soft tissue infection with male predisposition affecting the perineum and the genital area. We report, to our knowledge, the first case of Fournier’s gangrene caused by *C. ramosum* in a female patient with multiple underlying conditions.

**Case presentation:**

A 44-year-old woman with a 6-year history of insulin-dependent diabetes mellitus after total pancreatectomy and an 11-year history of central diabetes insipidus developed a pain in the genital area after a month of urinary catheter use. The lower abdominal pain worsened gradually over 2 weeks, and the pain, general fatigue, and loss of appetite prompted the patient’s hospital admission. As she had severe edema in her pelvic and bilateral femoral areas, ceftriaxone was started empirically after collecting two sets of blood cultures. On hospital day 2, CT examination revealed the presence of necrotizing faciitis in the genital and pelvic areas, and the antibiotics were changed to a combination of meropenem, vancomycin, and clindamycin. Gram-positive cocci and gram-positive rods were isolated from blood cultures, which were finally identified as *Streptococcus constellatus* and *C. ramosum* using superoxide dismutase and 16S rDNA sequencing. An emergent surgery was performed on hospital day 2 to remove the affected tissue. Despite undergoing debridement and receiving combined antimicrobial chemotherapies, the patient’s clinical improvement remained limited. The patient’s condition continued to deteriorate, and she eventually died on hospital day 8. In the present case, the underlying diabetes mellitus, urinary incontinence due to central diabetes insipidus, undernutrition, and edema served as the predisposing conditions.

**Conclusions:**

*C. ramosum* is a potentially opportunistic pathogen among immunosuppressed persons and a rare cause of necrotizing fasciitis.

## Background

*Clostridium ramosum* belongs to the genus *Clostridium*, a group of Gram-positive, spore-forming obligate anaerobes, currently known as *Erysipelactoclostridium* [1]. *C. ramosum* is a component of the normal intestinal flora [2] and has been rarely recognized as an etiologic agent of infectious diseases when isolated from clinical specimens [3].

Fournier’s gangrene is a form of necrotizing fasciitis or gangrene affecting the genital area. Shock and organ failure based on bacteremia frequently accompany gas gangrene, and the mortality rate is reported to be high around 50% in classical reports [4]. The most common pathogens of Fournier’s gangrene include *Escherichia coli* and *Bacteroides*. *Clostridium* species have been reported as the pathogen in approximately 10% of the cases of Fournier’s gangrene [5]. To date, only one case of gas gangrene caused by *C. ramosum* has been reported in the literature [6]. In the previous case reported in 1999, gas gangrene occurred in the neck and thorax, which is different from Fournier’s gangrene.

The underlying conditions help determine the prognosis of Fournier’s gangrene. Fournier’s gangrene commonly occurs in elderly men and rarely in women and is more likely associated with worse outcomes in individuals who are immunocompromised, such as those with diabetes. Treatments of patients with Fournier’s gangrene include the use of antibiotics and prompt surgical debridement. The diagnosis is suspected when gas in the deep tissue including the fascia is recognized by a computed tomography (CT) or magnetic resonance imaging (MRI) scan with systemic findings compatible with infection. It should be recognized as a condition that does not fall into the general category of “acute abdomen” but necessitates an urgent surgical referral.

We report a fatal case of Fournier’s gangrene caused by *C. ramosum* with *Streptococcus constellatus* in a patient with central diabetes insipidus and insulin-dependent diabetes mellitus. The infection had progressed for a few weeks and had subsequently rapidly worsened, taking a lethal course despite multimodal treatment.

## Case presentation

A 44-year-old Japanese woman with a 6-year history of insulin-dependent diabetes mellitus and an 11-year history of central diabetes insipidus presented with a pain in the genital area worsening over 2 weeks, general fatigue, and loss of appetite. Two months earlier, patient underwent a urinary catheter insertion as a management for urinary frequency, but it was removed during the previous hospital stay, a month before her recent hospitalization, for possible urinary infection. She has had frequent hospital admissions (6 times/year) and was hospitalized 3 months before her recent admission because of edema of the pelvic area and lower limbs. The patient developed diabetes mellitus after undergoing total pancreatectomy for nesidioblastosis, a surgical procedure which involved the removal of patient’s pancreas including the spleen and gallbladder. Her sister was also diagnosed with idiopathic central diabetes insipidus; thus, a family etiology was suspected. The patient had a surgery for suspected tongue cancer 2 years ago and was also suspected of non-alcoholic steatohepatitis with episodes of hepatic encephalopathy. Although she was on multiple medications including subcutaneous insulin injections and desmopressin tablets, her glycemic and hydration status were poorly controlled.

Four days prior to the present admission, she visited a gynecologist for her inguinal pain. No uterine tenderness or exudate was observed, and she was prescribed gentamicin and lidocaine ointments for possible local infection. She developed edema in the pelvic area with loss of appetite, and her home doctor consulted the university department 1 day before the present admission.

Upon admission, the patient appeared weak but was alert and had low-grade fever (37.4 °C) under a regular use of acetaminophen (1500 mg/day) and diclofenac (75 mg/day). Her blood pressure was not significantly different from previous measurements (88/42 mmHg) but a sinus tachycardia (heart rate 125/min) was noted. She complained of continuous abdominal pain and tenderness in all four quadrants. No abdominal guarding or rigidity was observed, but she had severe edema in the pelvic and bilateral femoral areas without necrotic skin discoloration. Laboratory investigations revealed a white cell count of 16,310/μL with neutrophilia (90.8%), elevated C-reactive protein of 22.18 mg/dL, and no serum sodium or potassium abnormalities. Serum aspartate aminotransferase and alanine aminotransferase were elevated at 466 U/L and 148 U/L, respectively. The patient’s international normalized ratio was high (2.26), but disseminated intravascular coagulation score did not meet the criteria. The patient’s HbA1c level was 8.8%, and blood sugar at admission was 316 mg/dL. She had low serum albumin concentration (1.7 g/dL), elevated serum ammonia concentration (154 μg/dL), and elevated lactate level (10.3 mmol/L). No ketonuria was noted, but significant pyuria was observed.

**Fig. 1:**
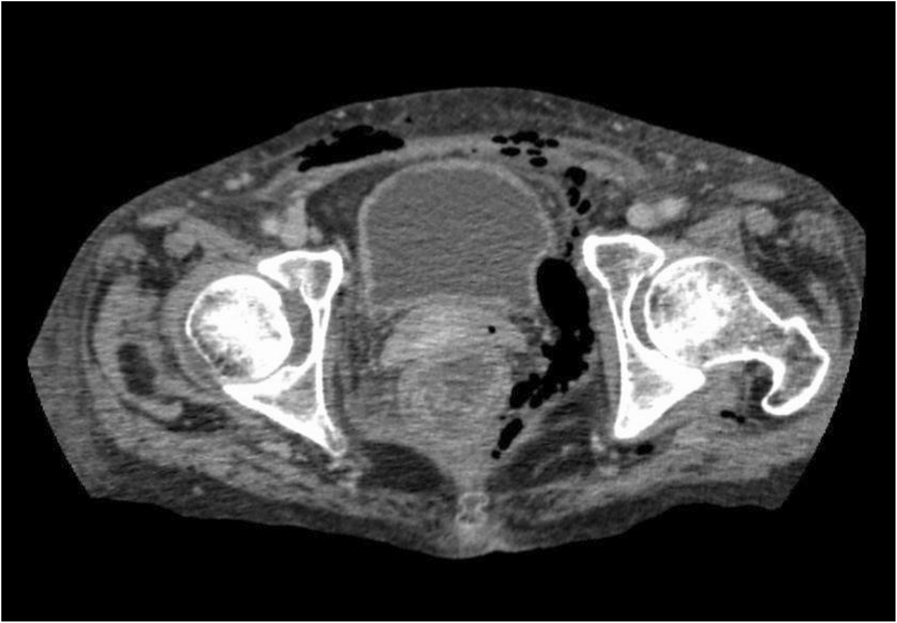
Computed tomography image of the lower-abdominal area before the surgery

The abdominal ultrasound was unrevealing; thus, an intravenous treatment with ceftriaxone (1 g every 8 h) was initiated empirically after obtaining the blood and urine culture samples. A CT scan performed the following morning revealed the presence of air in the soft tissue of the inguinal and pelvic areas, such as pectineal and psoas major muscles (Fig. 1). Immediate infectious and surgical consultations were made, and the antibiotics were changed to meropenem (1 g every 8 h), vancomycin (1 g every 12 h), and clindamycin (600 mg every 8 h). Gram-positive cocci and gram-positive rods were found in the initial blood cultures. In the evening of hospital day 2, a surgical debridement of the extraperitoneal pelvic tissue with colonostomy was performed, and the CT image after the operation suggested a complete resection of the affected tissue. However, hypernatremia (a serum Na concentration of 160 to 170 mEq/L) ensued as the use of nasal desmopressin could not effectively control the patient’s central diabetes insipidus after the operation.

No bacteria could be cultured from the debridement tissues (Table 1). The blood culture isolates were finally identified as *Streptococcus constellatus* using superoxide dismutase A sequencing and *C. ramosum* by 16S ribosomal DNA sequencing [7]. The minimum inhibitory concentrations (MICs) of various antibiotics were tested using Etest for *C. ramosum* [8] and the broth microdilution method [9] except imipenem and meropenem (Etest) for *S. constellatus* (Table 2). These results were interpreted using the Clinical & Laboratory Standards Institute M11-A8 document [10] for *C. ramosum* and M100-S24 document for *S. constellatus.* Both strains were susceptible to penicillin, meropenem, and clindamycin. Despite the continued use of susceptible antibiotics and intensive care, disseminated intravascular coagulation and pancytopenia developed, and the patient died on hospital day 8.

**Table 1:** Wound and other culture results

Source	Microbe	Quantity
Urine	*Klebsiella pneumoniae subsp. pneumoniae*	10^8
	*Staphylococcus aureus* (MSSA)	10^3
Wound swab in the genital area^a^	Negative	
Vaginal fluid^a^	Aerobic GPR	2+
	*Candida albicans*	2+
	Candida glabrata	few
	Fermentative GNR	few
	Staphylococcus aureus (MSSA)	few

^a^Samples taken after the start of antimicrobial agents

**Table 2:** Antimicrobial susceptibility profiles of *C. ramosum* and *S. constellatus* strains

Antibiotics	MIC (mg/L) and interpretation of susceptibility			
	*C. ramosum*		*S. constellatus*	
Benzylpenicillin	0.023	S	0.06	S
Ampicillin	0.047	S	ND	
Ampicillin/sulbactam	0.032	S	ND	
Piperacillin/tazobactam	0.094	S	ND	
Cefoxitin	64	R	ND	
Ceftriaxone	0.5	NA	0.5	S
Imipenem	0.25	S	0.094^a^	NA
Meropenem	1	S	0.125^a^	S
Clindamycin	0.75	S	≤0.125	S
Erythromycin	> 256	NA	≤0.125	S
Tetracycline	32	NA	0.5	S
Levofloxacin	> 32	NA	0.5	S
Moxifloxacin	> 32	R	ND	
Vancomycin	4	NA	1	S
Linezolid	4	NA	0.5	S
Metronidazole	0.75	S	ND	

*NA* not applicable, *ND* not done, *S* susceptible, *R* resistant

^a^ results using Etest

## Discussion and conclusions

Fournier’s gangrene is a very rare and severe infection affecting the soft tissues of the genital and pelvic areas. In a population-based study, the overall incidence was estimated to be 1.6 in 100,000 men, and it is expected to be much lower in women, as the study identified only 39 female patients as opposed to 1461 male patients [11]. However, the female ratio may be higher, as a recent study reported 20.2% female among 124 Founier’s gangrene patients [12]. Also, in a recent report, 9249 Fournier’s gangrene patients were identified for a weighted estimate of 43,146 cases using the National Inpatient Sample, and this study reported a mortality rate of 4.7%, which is lower than those of most previous reports [13].

Scoring systems have been proposed to establish a timely diagnosis and predict outcome of Fournier’s gangrene. One such system, Uludag Founier’s Gangrene Severity Index (UFGSI), evaluates the physiological status, age, and extent of the gangrene, and is reported to be more powerful than FGSI, another scoring system for Fournier’s gangrene that can predict mortality with a 78% probability and survival with a probability of 78% [14]. UFGSI score greater than 9 resulted in a 94% probability of death in contrast to a score of 9 or less that showed a high survival probability of 81% [15]. Our patient had FGSI score of 8 and UFGSI score of 14 upon admission, which increased to 12 and 18, respectively, at the time of debridement because electrolytes fluctuated due to central diabetes insipidus, and the prognosis was poor at that point.

In the present case, many possible underlying factors were identified: susceptibility to infection due to diabetes mellitus and undernutrition, the history of splenectomy, and compromised skin and mucosal barriers due to edema, incontinence, and urinary catheter placement for central diabetes insipidus. In addition, hypernatremia due to central diabetes insipidus complicated the postoperative management.

Moreover, the subacute onset of Fournier’s gangrene in this patient may have delayed the definite diagnosis. The presence of pain for 2 weeks suggested a less fulminant progression, but the condition was more severe internally than it seemed externally at presentation.

In the present case, the *C. ramosum* was isolated from the blood of a patient with Fournier’s gangrene for the first time in literature. *C. ramosum* cultures were also found in children with ear infections and in older adults with co-morbidities, sometimes forming abscesses [16]. The identification of *C. ramosum* in culture may be difficult: *C. ramosum* has a variability in Gram stain positivity, weak spore formation, and atypical clostridial colony morphology [17]. Molecular identification should be considered to identify suspected species of *C. ramosum*.

As for the pathogenic potential of *C. ramosum*, IgA protease of *C. ramosum* is able to degrade both IgA1 and IgA2, which helps the evasion of the microbe from the host immune defense to penetrate in to the deep tissue [2]. This IgA protease, cloned in 2002 [18], is not found in other *Clostridium* species. Because *C. ramosum* is among the normal intestinal flora, *C. ramosum* in this case may have entered the deep tissue of the genitalia via the intestinal mucosa. It is difficult to assess the pathogenic role of *C. ramusum*, but the blood cultures also yielded *S. constellatus,* a part of the *S. anginosus* group (formerly “*S. milleri*”) that causes various abscesses in humans and is often involved in polymicrobial infections with obligate anaerobes [19]. Anaerobes can enhance the growth of *S. constellatus* and inhibit the bactericidal activity of the host [20, 21]. In this case, the severity of the clinical course might be due to the synergistic infection of the *C. ramosum* and *S. constellatus*. Although antibiotic resistance is uncommon in *C. ramosum*, a 74% resistance to clindamycin was reported in one study [3]. In our case, this *C. ramosum* strain showed resistance to moxifloxacin and cefoxitin, but was susceptible to meropenem and clindamycin and showed low MIC of the initial antibiotic used, ceftriaxone. *S. constellatus* was also susceptible to ceftriaxone, meropenem, vancomycin, and clindamycin. The reason for the lethal course despite the use of multiple susceptible antibiotics combinations may be due to the uncontrollable progression of invasion with poor host-defense mechanism.

In conclusion, to our knowledge, we report the first case of Fournier’s gangrene with *C. ramosum* in a female patient with multiple comorbidities. The condition was more severe than it seemed at presentation, and the disease progressed rapidly despite multimodal treatment. The accumulation of more information on *C. ramosum* infections and gangrene may lead to the identification of the disease patterns and methods for timely diagnosis and treatment.

## Abbreviations

CT: Computed tomography
MIC: Minimum inhibitory concentration
